# Somatic pharmacogenomics of gastrointestinal stromal tumor

**DOI:** 10.20517/cdr.2019.02

**Published:** 2019-03-19

**Authors:** Gloria Ravegnini, Patrizia Hrelia, Sabrina Angelini

**Affiliations:** Department of Pharmacy and Biotechnology, University of Bologna, Bologna 40126, Italy.; ^#^These authors equally contributed to this work.

**Keywords:** Gastrointestinal stromal tumor, imatinib, pharmacogenomics, pharmacogenetics, tyrosine-kinase inhibitors

## Abstract

Gastrointestinal stromal tumors (GISTs) are rare entities, which, however, represent the most common mesenchymal tumor of the gastrointestinal tract. The discovery of gain of function mutations on *KIT* and *PDGFRA* receptor genes led to a deep revolution in the knowledge of this tumor. This paved the way to the introduction of imatinib and other tyrosine-kinase inhibitors (TKIs), which terrifically revolutionized the prognosis of GIST patients. Currently, it is well established that tumor mutational status is the main player in clinical outcome; however, with the research advances, it has been slowly understood that GIST landscape is more complex than expected and the TKIs available are not effective for all the GIST subtypes. For this reason, in the era of tailored/personalized medicine, each GIST patient should be considered individually and genetic consult should be the first step to take in consideration in the therapeutic decision making process.

## Introduction

Gastrointestinal stromal tumors (GISTs) are rare entities, which, however, represent the most common mesenchymal tumor of the gastrointestinal system^[1,2]^. Before the groundbreaking identification of activating mutations in the KIT tyrosine kinase receptor (*TKR*) gene in 1998^[3]^, GISTs were considered as a devastating disease due to scarce response to chemotherapy and radiotherapy. Luckily, the discovery of gain-of-function mutations on *KIT* and *PDGFRA* receptor genes led to a deep revolution in the knowledge of this tumor and from a poor characterized entity, GIST became a paradigm of target therapy. Indeed, at the beginning of 2000, the FDA approved the tyrosine kinase inhibitor (TKI), imatinib, for the management of metastatic and inoperable GISTs. Imatinib - the *magic bullet*, originally developed for chronic myeloid leukemia - is the first example of molecular-targeted drug with a known mechanism of efficacy; it represents the worldwide paradigm of targeted therapy specifically tuned *vs.* specific molecules - peculiar of the cancer cells^[4]^.

### GIST

After the identification of driver genetic events in 1998 and until a few years ago, GISTs were classically dichotomized in *KIT/PDGFRA* mutant (about 85%-90%), or *KIT/PDGFRA* wild-type (WT) GISTs or, often called, WT GISTs. WT GIST are a small group harboring a plethora of alterations on different genes, including succinate dehydrogenase (*SDH*), *NF1*, *BRAF*, *KRAS*^[5,6]^. However, with the fast advances in sequencing technologies, different studies have showed novel genetic mutations in the WT GISTs subgroup. It became clear that GISTs are a heterogeneous family of tumors, fragmented in different subtypes with specific and peculiar features^[7,8]^, which influence prognosis as well as clinical outcome. In 2014, a report from NIH suggested to classify GISTs in SDH competent, with characteristics in common with the classic *KIT/PDGFRA* mutant GISTs, and SDH-deficient^[9]^.

### SDH competent GISTs

#### KIT/PDGFRA mutation

As previously mentioned, the majority of GISTs harbor a mutation in *KIT* or *PDGFRA* genes. Specifically, approximately 80% of GISTs carry pathogenic activating mutations on *KIT*, whereas 5%-10% harbor *PDGFRA* mutations^[10,11]^. These mutually exclusive mutations are gain of function mutations, leading to a constitutively and ligand independent activation. This means that the receptors promote the activation of downstream pathways involved in many key biological processes of carcinogenesis, including RAS/RAF/MEK and PI3K/AKT/mTOR, and MAPK cascades^[12,13]^. Genetic alterations in *KIT* or *PDGFRA* genes - that can be simple aminoacid substitutions, in frame deletions or insertions - involve two main regions of the receptors, the regulatory domains and the enzymatic domains. Considering KIT receptor, the vast part of mutations (~65%) involve the exon 11, followed by 10% of cases who present a mutation on exon 9. In rare cases (~2%), primary *KIT* mutations can also hit exon 13 and exon 17. With regard to *PDGFRA*, the most common mutation (~5%) affects the exon 18 at codon 842, and promotes a substitution of an aspartic acid (D) with a valine (V) (D842V)^[14]^, while mutations on exon 12 and 14 are less frequent^[14]^. The main difference between *KIT* and *PDGFRA* mutation is the location within the receptor. Indeed, the majority of *KIT* mutations in GISTs arise in the juxtamembrane domain (exon 11), but only ~10% of *PDGFRA* mutations are in this region (exon 12). On the contrary, alterations within the activation loop of *KIT* (exon 17) are rare events (< 1%), but are prevailing in *PDGFRA*-mutant GISTs (exon 18)^[15]^.

#### BRAF/RAS and NF1 mutant GISTs

Among the SDH competent GISTs are included *BRAF/RAS* mutant and *NF1* mutant GISTs. GISTs with mutations in *BRAF/RAS* or *NF1* might be referred to as RAS-pathway mutant GISTs. It is estimated that among patients with no mutations on *KIT* or *PDGFRA* (*KIT*/*PDGFRA* WT), 5%-13% may have a genetic alteration on *BRAF*; in particular, > 90% of BRAF mutations occur in exon 15 on codon 600, usually V600E^[16-18]^*.* The V600E mutation promotes a BRAF activity due to creation of a salt bridge with the residue K507. V600E-K507 interaction mimics the conformational changes occurring during dimerization, so that BRAF V600E does not depend on dimerization for increased kinase activity^[19]^. BRAF is an intracellular protein kinase, playing a critical role in the RAS-RAF-MEK-ERK signaling pathway. Mutations on *KRAS* are events even more rare than BRAF ones; KRAS mutations in GISTs have a low frequency, spanning from ~1% to 11% of *KIT/PDGFRA* WT GIST^[16]^. Besides the mutations on *BRAF/RAS*, it has been reported that the autosomal-dominant inherited disease, neurofibromatosis Type 1 (NF1), promotes an increased incidence of GIST. In general, about 7% of cases with NF1 mutations will develop a GIST during their lifetime. This type of neurofibromatosis is characterized by genetic alterations on NF1 gene, which had more than 60 exons, and encodes neurofibromin, a tumor suppressor that downregulates the RAS/RAF/MEK/ERK pathway^[20]^.

### SDH deficient GISTs

The SDH deficient GISTs’ group includes GIST patients who lost the SDH complex functionality. Indeed, 10%-15% of adult GISTs do not harbor genetic alterations on *KIT* or *PDGFRA* but present alterations on one of the *SDH* genes. SDH is a mitochondrial enzyme composed by four different subunits, encoded by four different genes *SDHA*, *SDHB*, *SDHC*, and *SDHD*^[8]^. The SDH loss of expression is often due to germ-line and/or somatic loss-of-function mutations in any of the SDH subunits. In addition to the canonical DNA mutations, recently, different papers have showed that SDH inactivation may be due to epigenetic mechanisms as hypermethylation of *SDHC* promoter^[21]^. The SDH-complex is involved in the Krebs cycle and is responsible for the conversion of succinate to fumarate. Consequently, SDH deficiency leads to accumulation of succinate, which in turn promotes HIF1a overexpression and expression of hypoxia-associated tumorigenic responses and angiogenesis^[22]^. Considering the clinical features, SDH-deficient GISTs show a number of unique characteristics, such as young age at onset, female gender predilection, gastric localization, frequent lymph node metastatic involvement, and an indolent behavior^[7,23]^.

## Treatment of GIST

GIST management for immunohistologically confirmed GISTs plans: (1) surgical resection for resectable GISTs without metastasis, or (2) administration of TKIs for unresectable, metastatic, or recurrent GISTs^[24-26]^. Currently, the only first-line approved treatment for metastatic and inoperable GIST is imatinib. Imatinib, introduced in GIST management at the beginning of 2000, deeply changed the prognosis of these patients, who were considered irresponsive to the majority of available chemotherapic treatments^[27]^. Imatinib is a selective TKI, which targets diverse tyrosine kinase receptors, including ABL, BCR-ABL, KIT, PDGFRA, PDGFRB and CSF1R. The majority of GISTs respond well to the imatinib standard dose of 400 mg/day, but, commonly, after 24-36 months, a large proportion of patients develop secondary mutations and the tumor progresses^[28]^. To face the progressive acquisition of resistance, within the last 20 years, a second and a third line, sunitinib and regorafenib, respectively - have been introduced in GIST management. Sunitinib and regorafenib are TKIs^[29,30]^ with a wider range of kinase inhibition - compared to imatinib, including KIT, PDGFR, VEGFR, FLT3, TIE2, RET, FGFR1, RAF^[30,31]^. However, despite the efficacy of the currently available three-lines of therapy, patients usually progress even under sunitinib and regorafenib; unfortunately, there are no other therapeutic options and rechallenge of imatinib or sunitinib may represent a reasonable option in advanced GIST patients after failure of previous treatments^[32]^. In the last years, the research progress and the advance in deep sequencing techniques promoted the identification of novel potential targets and different trials are ongoing.

### KIT/PDGFRA genotype and clinical outcome

It is well established that tumor genotype play a critical role in GIST clinical outcome. Indeed, among GIST patients treated with TKIs, it has been observed a wide inter-individual variability and mutational analysis appear to be critical to make a clinical decision about adjuvant therapy.

Patients may show a primary resistance to the treatment (i.e., fail to respond within the first 6 months of treatment), or, as often happens in GISTs, stop to respond at some point, after an initial response (secondary or acquired resistance during treatment)^[33]^. It has been reported that about 10%-15% of GISTs treated with imatinib show primary resistance. Specifically, a meta-analysis of four studies involving 215 GIST patients evaluated resistance according to *KIT* and *PDGFRA* genetic alteration^[34]^. The authors found that 50% of *PDGFRA*-mutant, ~35% of *KIT/PDGFRA* wild-type and ~10% of *KIT* mutant GISTs were irresponsive to imatinib^[34]^. Considering the lack of an oncogenic mutation, it is not surprising the low imatinib response observed in *KIT/PDGFRA* WT GIST.

With regard to the other *KIT/PDGFRA* mutant GISTs, the mechanisms of primary resistance is quite unexpected. However, the most common *PDGFRA* mutation, D842V, is considered imatinib resistant; this is due to a conformational change located within the receptor activation loop which makes imatinib unable to bind it^[11]^.

Given the frequency, *KIT* mutant GIST patients have been studied more extensively than other subtypes.

Mutations on *KIT* exon 9, accounting for ~10% of GIST cases, are frequently characterized by duplications of six nucleotides (nt, encoding Ala-Tyr) at position 502-503, located within the extracellular domain. It is thought that the consequence of this duplication is an alteration in the receptor conformation, which mimics the binding of the physiological ligand, the stem cell factor, thus promoting constitutive activation^[8]^. *In vitro* studies have proven that mutations on *KIT* exon 9 reduce the sensitivity to imatinib^[35]^. Furthermore, presence of exon 9 mutations has been reported as the strongest adverse prognostic factor for imatinib response, and increases the relative risk of progression and death by 171% and 190% respectively, with respect to *KIT* exon 11 GISTs^[36]^. Results from different studies have shown that *KIT* exon 9 GISTs benefit from higher dose of imatinib, with significantly better progression-free survival (PFS)^[35,37,38]^. For this reason, this subset of patients is treated with 800 mg per day of imatinib, (instead of 400 mg), which is now considered the standard dose for this subgroup. In addition, for metastatic GISTs, it is strongly recommended that the treatment is continued indefinitely, as it has been observed that treatment outage is usually followed by quick tumor progression^[24]^. ESMO-EURACAN Clinical Practice Guidelines for diagnosis, treatment and follow-up released in 2018, highlight, once again, the importance of biopsy with histological and mutational analyses to propose the 800 mg imatinib dose for less sensitive *KIT* exon 9 mutations^[24]^.

*KIT* exon 11 GISTs are those patients who receive greater benefits from imatinib treatment compared with the other *KIT* mutational subtypes; indeed, these patients are twice as likely to respond to imatinib. In addition, *KIT* exon 11 show also higher response rate in terms of PFS and overall survival (OS). Primary mutations on *KIT* exon 13 characterize about 1% of GISTs; these genetic alterations promote changes within the receptors ATP binding pocket but the functional consequences have not been fully elucidate yet. The majority of data reported in literature show that mutations on *KIT* exon 13 confer sensitivity to imatinib, however a case report has described a rapid and aggressive tumor progression in a patient harboring a V654A alteration after imatinib and sunitinib treatment^[39]^.

With regard to *PDGFRA*, as mentioned before, carriers of D842V on exon 18 are usually primary resistant. *PDGFRA* D842V represents ~70% of all *PDGFRA* mutations in GISTs^[15]^. Besides D842V, patients may harbor other alterations on exon 18, which can hit close hotspots, as the aminoacids 843 to 845, and commonly are represented by deletions; in this case, GISTs are sensitive to imatinib. Currently, the largest study so far conducted, investigating 289 *PDGFRA* mutant GISTs, showed a variable grade of imatinib sensitivity basing on the specific mutation^[40]^. By virtue of these variable responses to imatinib observed in *PDGFRA* mutant GISTs, lately, different *PDGFRA* inhibitors have been entered into clinical studies. Among those showing better results, crenolanib was tested in a phase 2 trial and showed important preliminary clinical claims^[41]^; these data have contributed to the initiation of a phase 3 randomized, placebo-controlled trial of crenolanib activity in *PDGFRA* D842V-mutant GISTs (NCT02847429)^[42]^.

Table 1 summarizes correlation between exons harboring primary mutations and clinical response^[43,44]^.

**Table 1:** Gastrointestinal stromal tumor sensitivity to the approved tyrosine-kinase inhibitors basing on primary mutational status

Primary mutation	IM	SU	RE
KIT exon 9	S*	S	S
KIT exon 11	S	S	S
PDGFRA exon 12	S	S	U
PDGFRA exon 18 non D842V	S	S	U
PDGFRA exon 18 D842V	R	R	R

*800 mg instead of 400 mg/day. S: Sensitive; R: Resistant; U: Unknown; IM: imatinib; SU: sunitinib; RE: regorafenib

### Resistance mutations

As previously anticipated, progression after more than 6 months of initial clinical response is defined as secondary or acquired resistance. To date, secondary mutations have only been retrieved in patients with primary *KIT* mutations and rarely in those with primary *PDGFRA* mutations. Resistant mutations are most often found in the ATP-binding pocket of the kinase domain (exons 13 and 14) or in the kinase activation loop (exon 17 and 18)^[45]^. Despite the acquisition of secondary mutations, delayed resistance may be due to different mechanisms, including the KIT overexpression caused by genomic amplification, KIT loss of expression with activation of an alternative tyrosine kinase, and ABC transporters overexpression^[45]^. Therapeutic management of GIST patients progressed on imatinib consider dose escalation from 400 to 800 mg/day and/or switch to the second and third line TKIs.

The second line sunitinib inhibits several TKRs, blocking multiple biological processes, as tumor growth, angiogenesis and metastasis^[46]^. Sunitinib is effective in imatinib resistant GISTs and it has been shown that its activity is affected by the specific primary and secondary mutation^[47]^. In this context, exon 9 mutant patients usually have higher clinical benefits and objective response rates, compared with exon 11 mutants. In addition, both PFS and OS are generally significantly longer in *KIT* exon 9 mutant or *KIT/PDGFRA* WT GISTs with respect to *KIT* exon 11 mutant patients^[47]^. Taking into consideration the acquired mutations, sunitinib seems to be more effective in case of alterations within *KIT* exon 13 and 14 (ATP binding pocket) rather than those within KIT exon 17 or 18^[47]^.

Table 2 shows the correlation between the main secondary *KIT* mutations and sensitivity to approved TKIs.

**Table 2:** Gastrointestinal stromal tumor sensitivity to the approved tyrosine-kinase inhibitors basing on secondary mutations

Secondary KIT mutation	IM	SU	RE
KIT V654A	R	S	R
KIT T670I	R	S	R
KIT D816A/G/H/V	R	R	R
KIT D820A/E/G/Y	R	R	S
KIT N822H/K	R/S	R	S
KIT Y823F	R	R	S
KIT A829P	R/S	R	S

S: Sensitive; R: Resistant; R/S: intermediate sensitivity; IM: imatinib; SU: sunitinib; RE: regorafenib

#### SDH deficient GISTs

It is not surprising that *KIT/PDGFRA* WT GIST show poor response to imatinib and it has been reported that this GIST subset has a 76% higher risk of death if compared with *KIT* exon 11 mutants^[36]^. In this regard, the reports in literature agree in finding a very low benefit for these subsets of GISTs under imatinib treatment^[9]^. Furthermore, it seems that *KIT/PDGFRA* WT-SDH deficient GIST may have better response to sunitinib and regorafenib^[9,48]^. Sometimes, metastatic tumors can be quite indolent so that a “wait-and-see” policy applies, while other patients could benefit from resection. However, considering the complex molecular landscape of this subset, genetic counseling and therapeutic management plan in a GIST treatment center should be strongly taken into account.

#### BRAF/RAS and NF1 mutant GISTs

With regard to *BRAF* status, *in vitro* studies showed that alterations on *BRAF* gene confer imatinib resistance^[49]^ and, in general, to TKIs including sunitinib, regorafenib and sorafenib^[50,51]^. It has been proposed that this subset of GIST may have benefits from treatment with BRAF inhibitors, as dabrafenib, even if data are quite limited^[51]^.

The data on the prognosis of *NF1* mutant GISTs are even more scarce and controversial. A first report showed a general good overall response in a long term follow-up, with only 5 out of 35 patients who died due to metastatic disease. On the contrary, two following case reports described *NF1* mutant GISTs primary resistant or only initially responding to imatinib^[52-54]^. Currently, a Phase II Trial of evaluating MEK1/2 inhibitor, selumetinib, is ongoing for NF1 mutant GIST patients (NCT03109301).

## Germline DNA alterations in GIST

As reviewed, imatinib has represented a groundbreaking in GIST history. After its introduction, a huge research effort has been directed to the identification of driver mechanisms of acquired resistance, as well as, novel potential biomarkers for GIST treatment. In this context, the involvement of pharmacogenetic and epigenetic mechanisms in TKIs resistance has been investigated. In particular, with regard to pharmacogenetics, any drug goes through a specific pharmacokinetics itinerary that might be relevant for both drug efficacy and toxicity^[55,56]^. Therefore, it is conceivable that polymorphisms or gene expression regulation through methylation/miRNA mechanisms could represent key players in affecting the final clinical outcome. By virtue of this consideration, possible pharmacogenetic^[57-62]^ and epigenetic^[63-66]^ mechanisms of imatinib and sunitinib resistance have been broadly investigated in GISTs^[67-71]^. Despite the extensive research, data are controversial and to date none of them supports the use of pharmacogenetic or pharmacoepigenetic tests to optimize treatment outcome in GIST patients.

## Conclusion

This review aimed to point out the complex landscape of GISTs. This family of tumors is quite heterogeneous and any oncologist and clinical pharmacologist should consider every case individually, in order to deeply and accurately characterize the molecular events driving tumorigenis and treatment response. Currently, we know that GIST mutational status genotype has diverse impact on the therapeutic decision-making and this should be at the base of the clinical GIST management [Table 1]. For this reason, the tumor genetic consult should be crucial and strongly suggested by the oncologists who care the patients. However, in the era of personalized medicine, the unmet need for a precision medicine approach in GIST is more pivotal than ever. Indeed, although the currently available data points out that the tumor mutational status weighs more than the germinal one, the healthy patient’s DNA is still relevant. Therefore, even the currently available evidence do not allow translation of genetic test into routine clinical practice, implementation of pharmacokinetics, supported by trials, is needed.

## Declarations

### Authors’ contributions

Wrote the manuscript: Ravegnini G, Angelini S

Revised the manuscript: Hrelia P

### Availability of data and materials

Not applicable.

### Financial support and sponsorship

This work was supported by the Ministry of Education, University and Research of Italy (MIUR) (2015Y3C5KP_002) to Angelini S; the L’Oréal-UNESCO for Women and Science Award to Ravegnini G.

### Conflicts of interest

All authors declared that there are no conflicts of interest.

### Ethical approval and consent to participate

Not applicable.

### Consent for publication

Not applicable.

### Copyright

© The Author(s) 2019.
